# Characterizing Cold Days and Spells and Their Relationship with Cold-Related Mortality in Bangladesh

**DOI:** 10.3390/s23052832

**Published:** 2023-03-05

**Authors:** Md. Mahbub Alam, A. S. M. Mahtab, M. Razu Ahmed, Quazi K. Hassan

**Affiliations:** 1 Department of Physics, Khulna University of Engineering and Technology, Khulna 9203, Bangladesh; 2 Schulich School of Engineering, University of Calgary, Calgary, AB T2N 1N4, Canada

**Keywords:** air temperature, cold-related death or mortality, temperature anomaly, time series data analysis, severity of the cold days and spells

## Abstract

This research examined the characteristics of cold days and spells in Bangladesh using long-term averages (1971–2000) of maximum (*T_max_*) and minimum temperatures (*T_min_*) and their standard deviations (SD). Cold days and spells were calculated and their rate of change during the winter months (December–February) of 2000–2021 was quantified. In this research, a cold day was defined as when the daily maximum or minimum temperature is ≤−1.5 the standard deviations of the long-term daily average of maximum or minimum temperature and the daily average air temperature was equal to or below 17 °C. The results showed that the cold days were more in the west-northwestern regions and far less in the southern and southeastern regions. A gradual decrease in cold days and spells was found from the north and northwest towards the south and southeast. The highest number of cold spells (3.05 spells/year) was experienced in the northwest Rajshahi division and the lowest (1.70 spells/year) in the northeast Sylhet division. In general, the number of cold spells was found to be much higher in January than in the other two winter months. In the case of cold spell severity, Rangpur and Rajshahi divisions in the northwest experienced the highest number of extreme cold spells against the highest number of mild cold spells in the Barishal and Chattogram divisions in the south and southeast. While nine (out of twenty-nine) weather stations in the country showed significant trends in cold days in December, it was not significant on the seasonal scale. Adapting the proposed method would be useful in calculating cold days and spells to facilitate regional-focused mitigation and adaptation to minimize cold-related deaths.

## 1. Introduction

Cold days and spells are one of the natural hazards/disasters during the winter season in different parts of the world, including sub-tropical countries like Bangladesh in the northern hemisphere. The cold conditions are frequently associated with a rapid fall in air temperature, even within a day, as a result of: (i) the movement of the cold air mass, typically from the north or north-west, (ii) receiving less solar radiation, (iii) high wind speeds, and (iv) high humidity. In fact, Bangladesh is a South Asian country that experiences periodic episodes of cold days and spells in winter. Such cold conditions have devastating impacts on public health, agriculture, transportation, tourism, and overall socio-economy [1,2,3,4,5,6,7,8]. In the case of public health, cold spells may potentially cause the occurrence of cold-related diseases, including influenza, pneumonia, respiratory complications, asthma, cardiovascular diseases, and diarrhea [1,9,10]. Consequently, severe suffering and even death have been observed in seniors, infants, and homeless persons in particular [11,12,13]. In recent decades, the world has experienced an increment in both the frequency and intensity of extreme weather events, including cold spells in the face of climate change [14,15,16,17]. Countries located at both high latitudes and altitudes are usually well-equipped to combat these cold spells and their associated impacts [18]. However, sub-tropical countries like Bangladesh are relatively less prepared in this context [19]. Hence, it is imperative to study the cold days/spells regimes in Bangladesh to facilitate the better development of policies and adaptation strategies to fight against the adverse impact of cold days/spells.

In characterizing cold days and spells, one of the simplest and easiest methods is to use the air temperature-specific single threshold related to the daily minimum (*T_min_*), maximum (*T_max_*), or average (*T_avg_*) values [9,20,21,22]. The use of such a single threshold has several issues and is not applicable for the following cases: (i) a country/region over a large latitude range [22,23], as a higher latitude would experience relatively colder temperature regimes [24,25], (ii) heterogenous topography forces temperature variations, even over a narrow latitudinal extent [2], and (iii) parts of a country that have exposure to large water bodies [26]. Bangladesh has been using a single *T_min_* threshold (i.e., ≤10 °C) to define a cold day for the entire country, and three or more consecutive cold days (for at least two adjacent weather stations) in declaring a cold spell/wave [27,28]. In general, the northern and northwestern parts of Bangladesh exhibit relatively lower temperatures in comparison to the other parts of the country in winter, particularly the entire southern and southeastern parts exposed to the Bay of Bengal [1].

In addressing the abovementioned issues, one of the alternate methods is the use of standard deviation (*SD_long-term_*) of the long-term temperature average (*T_avg-long-term_*), that may potentially facilitate the evaluation of cold regimes in comparison to the site-specific climatic conditions. In this method, a cold day [29,30,31,32,33,34,35,36,37] could be defined by the following equation (Equation (1)).

(1) $${\left[\left(T_{x}-T_{avg-long-term-x}\right)\leq \;m\times SD_{long-term-x}\right]}_{i}$$

where, *x* is the measured air temperature, such as minimum, maximum, or average, *m* (=1 to 3, in most cases) is the multiplication factor of the standard deviation, and *i* is the day of interest. Furthermore, a cold spell could be declared if the condition of a cold day continues consecutively for at least 2 or more days. Some example cases of using the method are summarized in Table 1.

Our overall objective of this study was to adapt the temperature anomaly method to be implemented in Bangladesh, which was accomplished in three steps. Firstly, the daily *T_min_* and *T_max_* in conjunction with their respective long-term average and 1.5 SD values were used using Equation (1) to define a cold day. It was due to several factors, including: (i) *T_min_* is normally immediately observed before sunrise when most people are inside their residence, so it has little cold effect during winter; (ii) *T_max_* occurs (in most cases) in the afternoon when the sun is slightly inclined in the west and spans for so long that it has prolonged cold impact for the whole day. For instance, if *T_max_* is considered around 22 °C at a particular location, it should be below 20 °C for most of times of the day; and (iii) *T_avg_* might have significant biases if the temperature’s diurnal range is relatively longer than usual; e.g., if *T_min_* equals to 9 °C and the *T_max_* is 26 °C, then *T_avg_* may be inclined towards the *T_max_*. Secondly, it was considered and determined, for evaluating how the ‘determined cold days’ were linked with cold-related mortality, whether there would be any modification required. In this case, the cold-related mortality database of Bangladesh was chosen, which was investigated in our earlier research [1]. It was assumed that modification would be needed in the relatively warmer coastal region of the study area, where the cold-related mortality database demonstrated a small number of death occurrences. In this context, it was proposed to use *T_avg_* based on 3 hourly data available from recent decades; an example case of calculating *T_avg_* using 1/2, 1, and 3 hourly, and daily *T_max_* and *T_min_* is shown in the Supplementary Materials. Furthermore, *T_avg_* was used to categorize cold days into five classes; i.e., extreme, severe, very, moderate, and mild. Finally, cold spells were calculated from the number of cold days using the following criteria; i.e., if cold days consecutively persisted for at least 2 days over two or more adjacent weather stations. Additionally, trends in cold days and spells were analyzed using the Mann-Kendall (M-K) test [45] and Sen’s slope estimator (SSE) [46].

## 2. Materials and Methods

### 2.1. Study Area and Data Requirement

**Figure 1:**
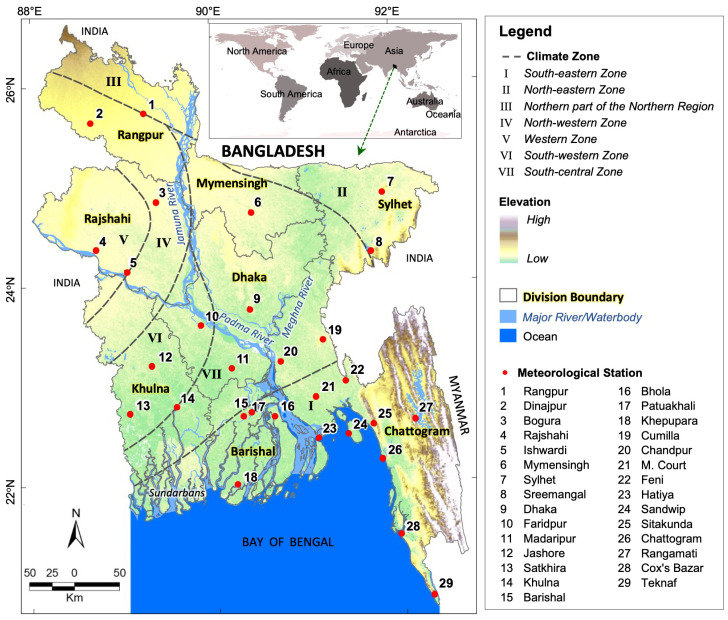
Demonstration of the study area with seven distinct climatic zones and eight administrative divisions (adopted from Alam et al. [1]).

Bangladesh is one of the South Asian countries with latitudes and longitudes spanning the range of 20°34′–26°38′ N and 88°01′–92°41′ E, respectively (see Figure 1), with an area coverage of 147,540 km^2^ [47]. It comprises eight administrative divisions, namely Rangpur, Rajshahi, Mymensingh, Sylhet, Dhaka, Khulna, Barishal, and Chattogram (see Figure 1). In terms of topography, the country exhibits mostly plain land with an average elevation of about 12 m from mean sea level, where the northern region is around 105 m, the northeastern region is 60–150 m [48], and the southeastern hilly region is about 600–1200 m above sea level [49]. Additionally, major features in the vicinity of the country include the Himalayas in the north and the Bay of Bengal in the south. On the basis of climate, Bangladesh is categorized into four distinct seasons, including the summer season between March and May, the monsoon season between June and September, the autumn/fall seasons between October and November, and the winter season between December and February [28]. In general, Bangladesh experiences tropical climatic conditions that consist of seven sub-climatic zones; i.e., north-northern, northwestern, western, north-eastern, south-central, south-western, and south-eastern [1] (see Figure 1). The country observes an average precipitation of about 2400 mm/year [50], where a significant amount of precipitation (about 71%) takes place during the monsoon season [28]. In terms of temperature, the country has an annual average temperature of around 25 °C, while winter has an average between 17 and 20.6 °C [51].

In this study, two types of data were acquired. Firstly, the temperature data were collected from Bangladesh Meteorological Department (BMD) at 29 meteorological stations across Bangladesh (see Figure 1). For example: daily *T_min_* and *T_max_* were collected over the period 1971–2021 for most of the stations, except Feni and Patuakhali (1973–2021), and Khepupara (1975–2021). In addition, 3-hourly air temperature (*T_a_*) data were collected over the period 2000–2021. Secondly, the divisional cold-related mortality database was adopted for the winter months for the periods of 2009–2010 to 2020–2021, available in Alam et al [1]. The database was developed using online Bangladeshi national newspapers published during the period December 2009 to February 2021, which revealed that around 53.6% of deaths occurred in January (the coldest month) out of 1249 deaths during the study period.

### 2.2. Methods

**Figure 2:**
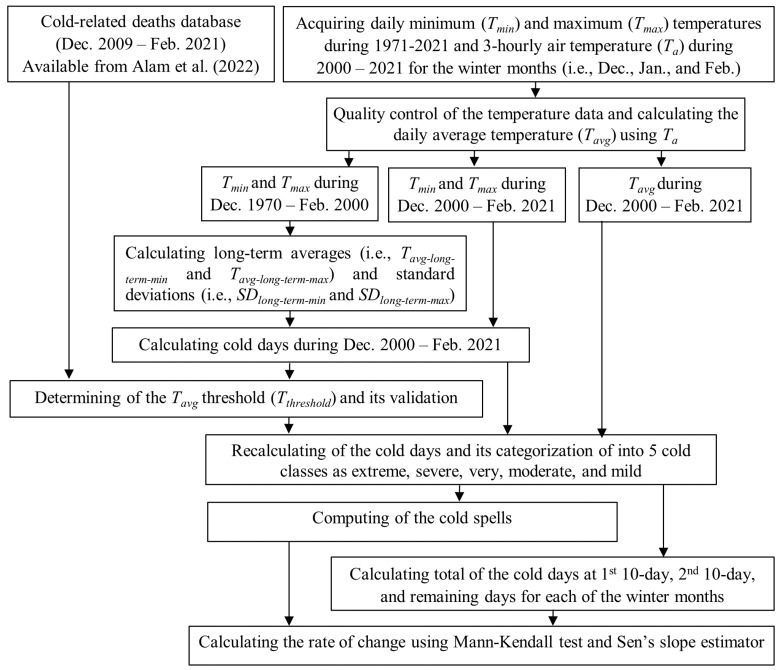
A schematic diagram illustrates the concept of developing methods for characterizing cold days and spells/waves [1].

Figure 2 shows the proposed methods implemented in this study. It comprised three components, such as (i) quality control of the temperature data, (ii) calculating cold days and spells, and (iii) quantifying the rate of changes in cold days and spells. All the components are briefly described in the subsequent sub-sections.

#### 2.2.1. Quality Control of the Temperature Data

Upon acquisition of the required temperature data, as described in Section 2.1, the dataset was examined to identify and remove the outliers using the following criteria:

- Whether the daily *T_min_* was higher than the daily *T_max_* at a given station of interest,
- If any of the values related to *T_min_*, *T_max_*, *T_a_* were less than zero, and
- If there were abrupt change(s) in any of the *T_min_*, *T_max_*, and *T_a_* values between successive days at a station of interest compared to its adjacent stations.

Moreover, data at the Chattogram station was missing during 2003–2007 due to rebuilding activities. Consequently, this data gap was filled using data from a nearby station, known as Chattogram Ambagan, located within a 9 km distance. About 4.83% of data of the total data was not available for this study.

#### 2.2.2. Calculating Cold Days and Spells

In the initial calculation of cold days, the temperature anomalies method was adapted using a multiplier of 1.5 in Equation (1). In this case, daily *T_min_* and *T_max_* were used to calculate their respective long-term averages (i.e., *T_avg-long-term-min_* and *T_avg-long-term-max_*) and standard deviations (i.e., *SD_long-term-min_* and *SD_long-term-max_*) over the period from December 1970 to February 2000 for the 29 meteorological stations at an everyday scale. Then, a cold day was computed for a given day (*i*) during the period from December 2000 to February 2021, if the following condition was met:

(2) $$\begin{array}{c} {\left[\left(T_{min}-T_{avg-long-term-min}\right)\leq \;1.5\times SD_{long-term-min}\right]}_{i}\\ or\\ {\left[\left(T_{max}-T_{avg-long-term-max}\right)\leq \;1.5\times SD_{long-term-max}\right]}_{i} \end{array}$$

**Figure 3:**
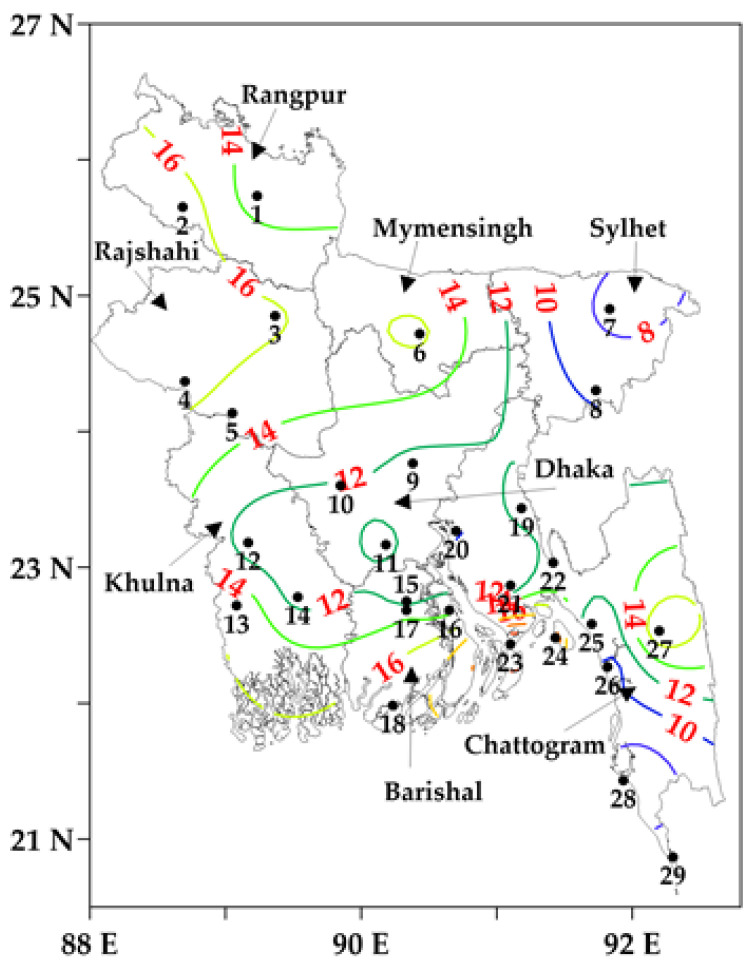
The initial calculation of the station-wise average cold days (in coloured contour lines with values) during the winter period from 2000–2001 to 2020–2021 across Bangladesh. The black numbers are the IDs of meteorological stations.

An initial assessment of the determined cold days revealed that the weather stations located adjacent to the coastline (i.e., the southern part of Bangladesh) had a relatively higher number of cold days (see Figure 3 for details). Thus, the definition of cold days was refined using daily *T_avg_* computed from 3-hourly *T_a_*-values. In this case, the most important issue was the determination of the *T_avg_* threshold (*T_threshold_*). Consequently, the impact of *T_threshold_* starting from 13 °C to 21 °C at 1 °C intervals was investigated using the death-related calibration database (i.e., winters from 2009–2010 to 2014–2015, and odd winters from 2009–2010 to 2020–2021). However, the *T_threshold_* was considered optimal when at least 80% of the deaths were observed. The optimal *T_threshold_* was applied using the death-related validation database (i.e., winters from 2015–2016 to 2020–2021, and even winters from 2009–2010 to 2020–2021) to determine its applicability. Note that the concept of using odd and even year data for calibration and validation was widely found in the literature [52,53] as it employs an equal amount of data in both calibration and validation phases.

In addition, cold days were recalculated by combining Equation (2) and *T_threshold_*, and were further categorized into the following arbitrarily defined five classes as follows: (i) extreme [≤*T_threshold_* −4 °C]; (ii) severe [≤*T_threshold_* −3 °C]; (iii) very [≤*T_threshold_* −2 °C]; (iv) moderate [≤*T_threshold_* −1 °C]; and (v) mild [≤*T_threshold_* °C]. Finally, cold spells could be declared if the condition continued for two or more nearby stations for at least two or more consecutive days.

#### 2.2.3. Quantifying the Rate of Changes in Cold Days and Spells

Upon calculating cold days and spells, the M–K test [45] and SSE [46] were applied to determine the rate of changes in both cold days and spells. The 10-day, monthly, and seasonal scales were considered for calculating cold days. For the 10-day scale, the first, second, and remaining days for each month in the winter season were considered. In the case of cold spells, the monthly and seasonal scales were considered for each administrative division, as spells would not normally be restricted to over a 10-day period. In the case of the M-K test, the S statistics were computed using Equation (3).

(3) $$S=\sum_{k=1}^{n-1}\sum_{j=k+1}^{n}\mathrm{sgn}\left(x_{j}-x_{k}\right)$$

where

(4) $$\mathrm{sgn}\left(x_{j}-x_{k}\right)=\{\begin{array}{c} +1\;if\;\left(x_{j}-x_{k}\right)>0\\ \;0\;if\;\left(x_{j}-x_{k}\right)=0\\ -1\;if\;\left(x_{j}-x_{k}\right)<0 \end{array}$$

*n* is the number of observations, and *x_j_* and *x_k_* are from *k* = 1, 2, …, n − 1 and *j* = *k* + 1, …, *n* in cold days and spells in the time series. The average of *S* is 0, and the variance of *S* is calculated using Equation (5).

(5) $$var\left(S\right)=\frac{n\left(n-1\right)\left(2n+5\right)}{18}$$

Furthermore, *Z* statistics with significance levels of 90, 95, and 99% confidence were employed. When *n* > 10, the *Z* is computed using Equation (6), where the positive and negative values indicate the increasing and decreasing rate of changes, respectively.

(6) $$Z=\{\begin{array}{c} \frac{S-1}{\sqrt{var\left(S\right)}}\;if\;S>0\\ \;0\;if\;S=0\\ \frac{S+1}{\sqrt{var\left(S\right)}}\;if\;S<0 \end{array}$$

The SSE was used to calculate the magnitudes of the rate of changes in cold days and spells using Equation (7).

(7) $$\beta =Median\left(\frac{x_{j}-x_{i}}{j-i}\right),\;j>i$$

where *β* is the Sen’s slope.

## 3. Results

### 3.1. Initial Calculation of Cold Days

Figure 3 represents the initial estimates of the station-wise average number of cold days during the winters of 2000–2001 to 2020–2021 across Bangladesh. The number of average cold days was found to be higher at all the stations except Rangpur (12.9 days/year) over the northwestern and northern divisions of Rangpur, Rajshahi, and Mymensingh in comparison to the country average (~13.2 days/year). Additionally, the four topmost average cold days were found in the southern and southeastern regional stations, namely Hatiya (24.7 days/year), Sandwip (21.7 days/year), Rangamati (18.1 days/year), and Khepupara (17.4 days/year). On the other hand, the four lowest average cold days were noticed in the southeastern and eastern regional stations, namely at Cox’s Bazaar (5.71 days/year), Sylhet (7.29 days/year), Chattogram (9 days/year), and Teknaf (9.14 days/year). In general, it has been found that the number of average cold days was relatively high at some coastal stations in the southern part of the study area.

### 3.2. Determining the Daily Average Temperature Threshold (T_threshold_) and Its Validation

Table 2 shows the percentage of cold-related deaths in the administrative divisions upon applying the *T_avg_*-values to both calibration and validation periods. In the calibration phase, it was found that 100% of mortalities in all divisions were when *T_avg_* was ≤21 °C. The mortality prominently varied in each 1 °C temperature interval between 13 to 21 °C in all of the divisions. Furthermore, an average mortality above 70% was observed for all the divisions when *T_avg_* ≤ 16 °C. Consequently, the *T_threshold_* was considered at least 16 °C in this research in defining a cold day. In the validation phase, observations showed that the *T_threshold_*-value of 17 °C was able to capture greater than an average of 70% of the deaths in all the divisions, not the 16 °C revealed in the calibration phase. Thus, the *T_threshold_*-value of 17 °C was chosen as the optimal one in this study for defining cold days.

### 3.3. Final Calculation of Cold Days and Classification

**Figure 4:**
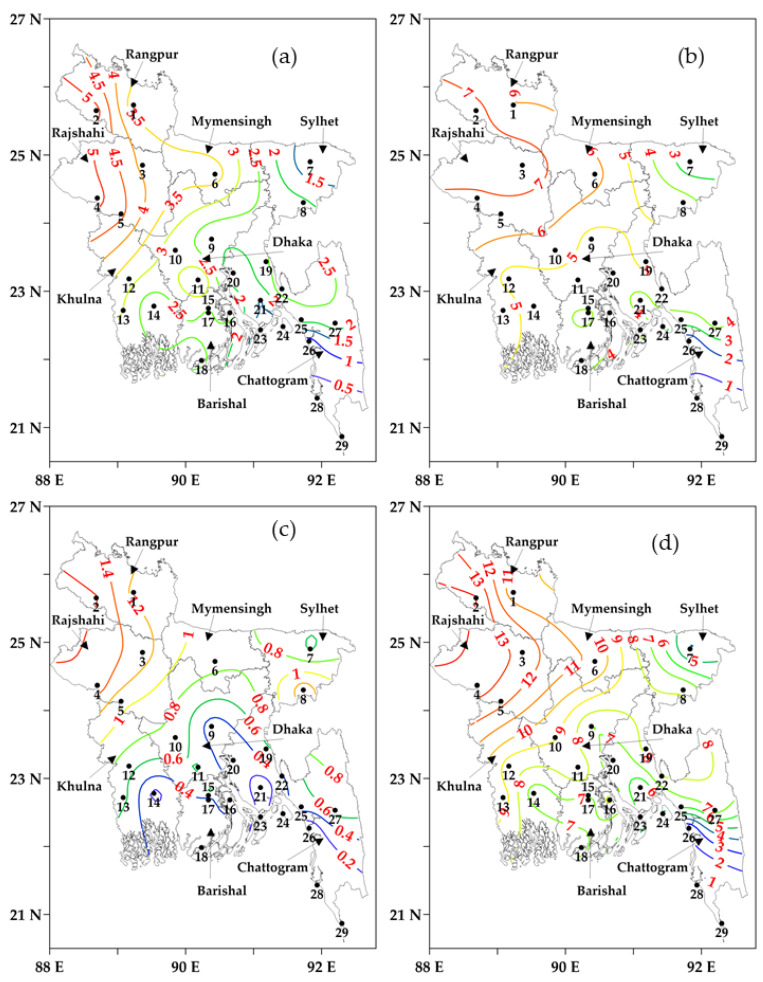
Calculation of the station-wise average cold days (in coloured contour lines with values) using the threshold of average temperatures, (**a**) December, (**b**) January, (**c**) February, and (**d**) the winter season during 2000–2001 to 2020–2021. The black numbers are the IDs of meteorological stations.

Figure 4a–d shows the final count of the station-wise average number of cold days upon applying the *T_threshold_* value of 17 °C during the winter periods 2000–2001 to 2020–2021 across Bangladesh. Figure 4a–d shows the final number of average cold days in December, January, February, and the winter season, which were much reduced in the southern and southeastern coastal stations after the application of average temperature threshold values. It revealed that the application of the *T_threshold_* had minimal effect on the west-northwestern stations. The number of cold days was found to be higher in the west and northwestern Rajshahi and Rangpur divisions and gradually decreased towards the south, southeast, and eastern regions of the country. The average number of cold days was found to be 4–5 days/year in December (see Figure 4a), 6–8 days/year in January (see Figure 4b), 1–1.6 days/year in February (see Figure 4c), and 11–14 days/year in the winter season in the west northwestern regions. On the other hand, the number of average cold days was found to be 0–2, 1–4, 0–0.5, and 1–7 days/year in December, January, February, and the winter season, respectively, at the south-southeastern coastal stations. The lowest average cold days were found at Teknaf (0.05 days/year) and Cox’s Bazar stations (0.71 days/year). In the case of divisions, the average number of cold days were: (i) 4.2, 4.5, 3.8, 1.6, 2.8, 2.8, 2.6, and 1.5 days/year in December; (ii) 6.6, 7.0, 6.3, 3.4, 5.0, 5.0, 4.3, and 3.2 days/year in January; and (iii) 12.2, 12.9, 11.0, 5.9, 8.3, 8.2, 7.4, and 5.0 days/year in the winter at Rangpur, Rajshahi, Mymensingh, Sylhet, Dhaka, Khulna, Barishal, and Chattogram, respectively. Additionally, it showed that the country’s average cold days were 2.48, 4.47, 0.59, and 7.55 days/year in December, January, February, and the winter season, respectively. In general, it was found that the average number of cold days was higher in January and lower in February, in the case of all stations. Furthermore, the cold days showed a gradual decrease from the northern and northwestern to the southern and southeastern regions of the country, except for Sylhet station.

**Figure 5:**
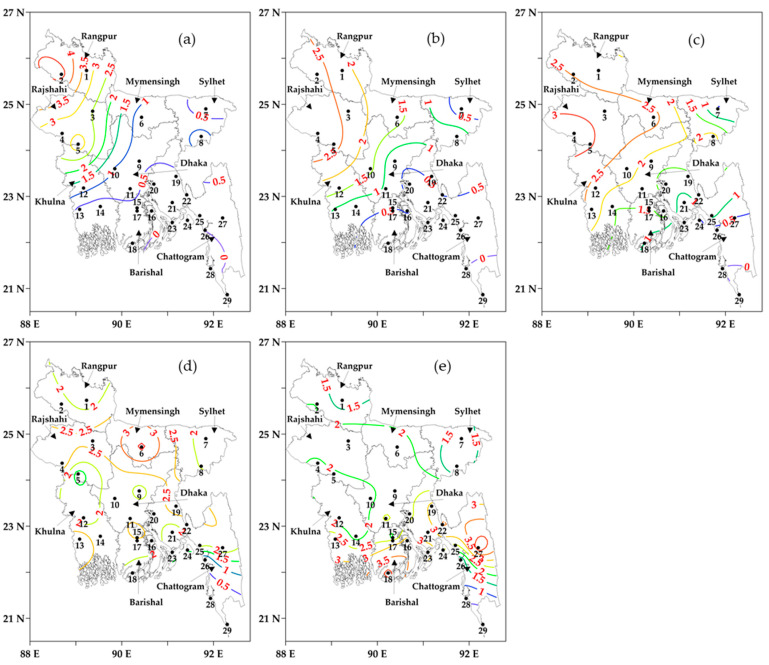
The categorization of average cold days (in coloured contour lines with values) using the threshold of average temperatures as: (**a**) Extreme, (**b**) Severe, (**c**) Very, (**d**) Moderate, and (**e**) Mild during the winters of 2000–2001 to 2020–2021. The black numbers are the IDs of meteorological stations.

Figure 5a–e represents the number of average cold days in different categories, such as extreme, severe, very, moderate, and mild, during the winters of 2000–2001 to 2020–2021 at all the weather stations across Bangladesh. It reported that the highest average extreme cold days were found at Dinajpur (4.7 days/year) and Ishwardi (3.4 days/year) in the northwest, while there were no extreme cold days observed at the southeastern coastal stations of Hatiya, Sandwip, Chattogram, Cox’s Bazar, and Teknaf. The number of average extreme cold days (see Figure 5a) was found to be 2–4.5 days/year, severe cold days 2–2.5 days/year (see Figure 5b), and very cold days 2.4–3 days/year (see Figure 5c) in the west-northwestern regions of the country. On the other hand, the number of average moderate cold days (see Figure 5d) was found to be 0–2.5 days/year and mild cold days were found to be 0–3.5 days/year (see Figure 5e) in the south-southeastern coastal stations. Furthermore, at the division level, Rangpur, Rajshahi, Dhaka, and Mymensingh divisions experienced extreme cold days of 3.98, 2.78, 0.79, and 0.71 days/year, respectively, and severe cold days of 2.43, 2.65, 1.05, and 1.67 days/year, respectively, on average. Furthermore, Rajshahi, Mymensingh, Rangpur, Khulna, Dhaka, and Barishal divisions exhibited very cold days of 3.02, 2.67, 2.31, 2.1, 1.97, and 1.45 days/year (on average), respectively. In this research, moderate cold days were observed in the range of 1.6 to 3.62 days/year, applicable across the divisions. Moreover, the coastal divisions, i.e., Barishal (2.99 days/year) and Chattogram (2.36 days/year), experienced the highest number of average mild cold days in comparison to the other divisions. In general, the extreme, severe, and very cold days were higher in the western and northwestern stations and gradually decreased from the west-northwest to the northeast, south, and southeastern regions of the country. Mild and moderate cold days were found to be higher in the southern and southeastern coastal stations of the country.

### 3.4. Computation of Cold Spells

**Figure 6:**
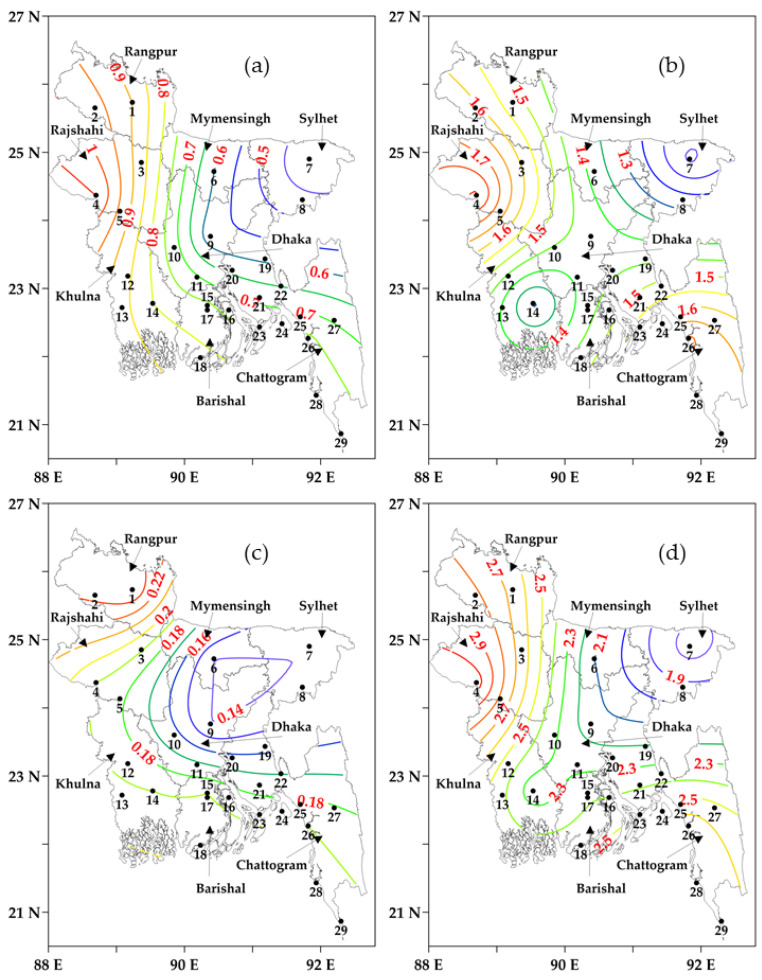
The average number of cold spells (in coloured contour lines with values) in (**a**) December, (**b**) January, (**c**) February, and (**d**) Winter in different divisions of Bangladesh during the winters of 2000–2001 to 2020–2021. The black numbers are the IDs of meteorological stations.

Figure 6a–d shows the number of average cold spells during the winters of 2000–2001 to 2020–2021 in all the divisions across Bangladesh. The number of average cold spells was higher in December (see Figure 6a), January (see Figure 6b), February (see Figure 6c), and in winter (see Figure 6d) at the west and northwestern Rajshahi and Rangpur divisions and gradually decreased towards the eastern Sylhet division. On average, the Rajshahi division experienced the highest number of cold spells (3.05 spells/year), and the Sylhet division had the lowest (1.70 spells/year) in winter. On a monthly scale, on average, Rangpur, Rajshahi, Mymensingh, Sylhet, Dhaka, Khulna, Barishal, and Chattogram divisions experienced 0.90, 1.05, 0.60, 0.40, 0.57, 0.80, 0.76, and 0.76 spells/year in December, respectively, 1.52, 1.81, 1.38, 1.14, 1.43, 1.29, 1.43, and 1.71 spells/year in January, respectively, and in the range of 0.15 to 0.25 spells/year in February. In general, the month of January had a much higher number of cold spells in comparison to the other two months in the winter season.

**Figure 7:**
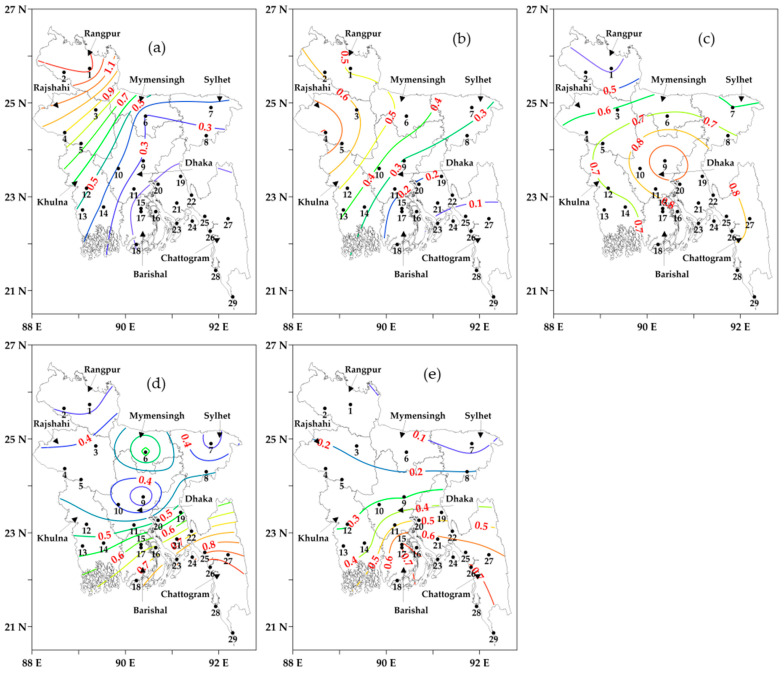
The average number of cold spells (in coloured contour lines with values) in (**a**) Extreme, (**b**) Severe, (**c**) Very, (**d**) Moderate, and (**e**) Mild in different divisions of Bangladesh during the winters of 2000–2001 to 2020–2021. The black numbers are the IDs of meteorological stations.

Figure 7a–e shows the number of different categories of average cold spells during the winters of 2000–2001 to 2020–2021 all over Bangladesh. The number of average extremes (see Figure 7a), severe (see Figure 7b), and very (see Figure 7c) cold spells was higher in the northwestern, western, and central regions, respectively. On the other hand, moderate (see Figure 7d) and mild (see Figure 7e) cold spells were higher in the southern and southeastern regions. In terms of the severity of cold spells, the northwestern regions (Rangpur and Rajshahi divisions with 1.33 and 0.90 spells/year, respectively) showed the highest number of extreme cold spells with a decreasing tendency towards the southern and southeastern regions (Barishal and Chattogram divisions with ~0.14 spells/year) in the coastal area. Mild cold spells had the highest number of occurrences in the coastal area (0.71 spells/year in both Barishal and Chattogram divisions). Additionally, Rangpur (1.81 spells/year) and Rajshahi (1.71 spells/year) divisions experienced the highest number of combined extreme and severe cold spells, on average, during the study period.

### 3.5. Trend Analysis of Cold Days and Spells

**Figure 8:**
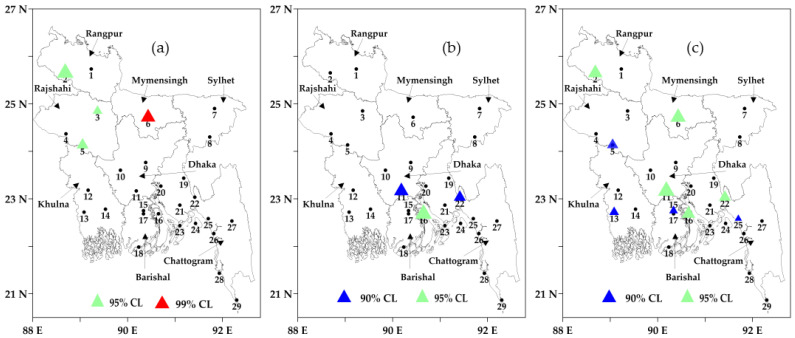
Significant decadal trends of the cold days during (**a**) 10–20 December, (**b**) 21–31 December, and (**c**) month of December as a whole across the country during 2000–01 to 2020–21, where CL means confidence level and the size of the triangle represents the magnitude of the decadal trend values. The black numbers are the IDs of meteorological stations.

The trend analysis of cold days at the 10-day scale showed that two of the total nine 10-day periods (11–20 December and 21–31 December) had significant trends at ≥90 confidence level (see Figure 8). During the 11–20 December period (see Figure 8a), four (all in the northern and northwestern regions) out of the twenty-nine stations showed significant increasing trends in the range of 0.28 to 1.76 days/decade. Regarding another period (21–31 December), during 21–31 December (see Figure 8b), three (all in the southern region) stations revealed increasing trends in the range of 1.18 to 1.6 days/decade. In the case of monthly and seasonal scales, it was observed that only nine stations had significant trends at a ≥90 confidence level in December (see Figure 8c). All these stations showed increasing trends in the range of 0.54 to 3.08 days/decade. The seasonal scale trend analysis demonstrated that there was no significant trend in the administrative divisions. In the case of cold spells, a significant trend was observed in December in the Barishal (0.28 spells/decade) division. Again, the categorization of cold spells showed that the trend was significant in the case of very cold spells at the Dhaka (0.65 spells/decade) division.

## 4. Discussion

**Figure 9:**
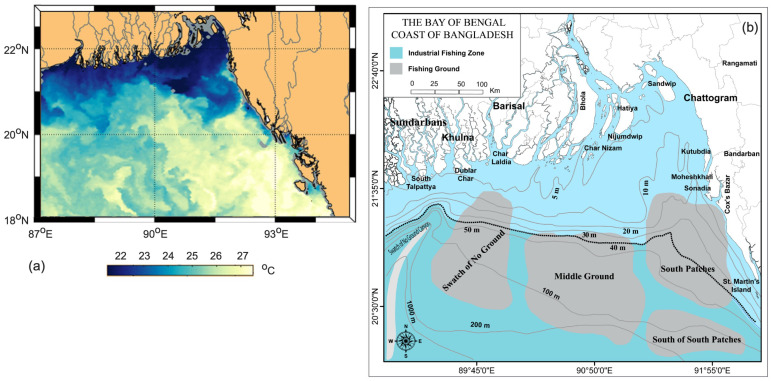
Dynamics of: (**a**) winter sea surface temperature [adapted from NASA], and (**b**) contour of the depth of the Bay of Bengal (adopted from © Alam et al, [55]) licensed under CC BY.

Upon completion of the initial calculation of cold days, a generic pattern of cold days was observed across the country, i.e., the annual average number of cold days decreased from the northern and northwestern towards the southern and southeastern regions (see Figure 3). However, the number of average cold days was relatively high at some coastal stations, including Hatiya and Sandwip, even though the coastal area is located in the southern part of the study area. In fact, it was observed in another study [54] that the percentage of cold days, defined as a function of *T_min_* (i.e., less than the 10th percentile), increased at some coastal stations, including the above two stations during 1968–2018. This emerged due to the fact that these two stations have shallow water compared to other coastal stations like Chattogram and Cox’s Bazar. It is a well-known fact that a greater depth of water slowly releases heat compared to shallow water, which is evident in Figure 9; thus, this would potentially influence the surrounding air temperature. Additionally, another coastal station, i.e., Rangamati, exhibited a higher number of cold days due to its relatively higher elevation in comparison to the other stations. Another study [28] also reported that *T_min-long-term_* had decreased at the station between the periods of 1971–2000 and 1981–2010.

Due to the overestimation of cold days, *T_avg_* was chosen as another variable to analyze the empirical relationships with cold-related mortality. In this study, the *T_threshold_* value of 17 °C was selected to coincide with at least 80% of the cold-related deaths across the country, except in Sylhet (47%) and Mymensingh (67%) divisions in the validation phase. In the case of the Sylhet division, the fewer deaths might be related to the fact that it has the best socio-economic conditions in the country [1]. Furthermore, the lower death percentage in the Mymensingh division could be due to cold-related critical patients transported to Dhaka City [56]. In fact, Dhaka has the best medical facilities in the country, and its distance from the Mymensingh division is relatively short. Finally, it would be worthwhile to note that it is not possible to compare the optimal *T_threshold_*-value of 17 °C as similar studies were not found in the literature.

The number of cold days, using the *T_threshold_* value, implicitly reduced in the southern coastal stations (see Figure 4). As a result, the number of cold days was observed to be higher in the western and northwestern regions and continuously decreased proceeding further to the southern and southeastern regions. Note that the abovementioned patterns could be attributed to the following reasons. Firstly, the northern and northwestern regions receive less solar radiation in comparison to the southern part of Bangladesh, a common case in the Northern Hemisphere [24,25]. Secondly, during the winter season, cold air from the Himalayas enters Bangladesh through the northwest and western regions of Bangladesh, before flowing throughout the country [27,57], also flowing to the northeastern region [27]. Again, the cold air extends eastwards from the Himalayas and enters at Sreemangal station in Bangladesh after being intercepted by the Tripura Hills. The northeasterly cold wind hits the Sreemangal station earlier than the Sylhet station; thus, Sreemangal usually experiences lower temperature regimes in the winter. Finally, the southern coastal stations are located in the vicinity of the Bay of Bengal (a large waterbody), which slowly cools in winter depending on the water depth, and keeps the surrounding air relatively warmer than other regions [58]. Additionally, the generic pattern of the cold spells was identical to the final cold days across the country, where the abovementioned reasons were also applicable in observing it. This research will help to develop further adaptation and mitigation during cold in the west-northwestern regions than in the southern and southeastern regions.

Though numerous studies examining the air temperature (minimum, maximum, and average) trends during the entire winter seasons of over at least 30 years in Bangladesh [54,59,60,61], in this research, cold days and spells were determined based on the daily dynamics of the temperature. A similar study was conducted by Baten et al. [57], which reported both the estimates of cold days and spells during the 1988–2017 period. However, it did not help us to compare our findings, because the method used was based on the exploitation of the absolute threshold of the *T_min_*, which was completely different in comparison with the proposed method. In addition, the trend analysis of cold days during 10–day periods, months, and in the winter season was conducted in this study. The increasing trend in cold days was only found to be significant at a few stations during the 10-day period of 11–20 and 21–31 December. On the monthly scale, a significant trend in cold days was observed at nine stations in December, and no significant trend was found in January and February, as well as the winter season as a whole. At the administrative division level, the trend in cold waves was found to be significant at Barishal in the month of December and, according to the categorization, it was significant for very cold waves in the Dhaka division. No trend was seen in other months and categories.

## 5. Concluding Remarks

The daily average of long-term maximum and minimum temperatures and their standard deviations during 1971–2000 were analyzed to calculate cold days and spells in Bangladesh during the winter months of 2000–2021 and the rate of changes in cold days and spells was quantified. A cold day was defined at a station if the following conditions were fulfilled: (1) the maximum temperature or minimum temperature of a particular station was 1.5 standard deviation below the long-term average of the respective temperatures, and (2) the average 3-hourly air temperature was equal to or less than 17 °C. On the basis of average air temperature, the cold days were classified into five classes, e.g., extreme (≤13 °C), severe (≥13 to 14 °C), very (≥14 to 15 °C), moderate (≥15 to 16 °C), and mild (≥16 to 17 °C). The number of cold days was found to be maximum in the north and northwestern divisions in Bangladesh during December (4–5 days/year), January (6–8 days/year), and February (1–1.6 days/year). It gradually decreased towards the southern and southeastern regions, with the lowest found in the coastal divisions, including very few exceptions. Extreme and severe cold days were mainly found in the northwestern and western regions. On the other hand, more mild and moderate cold days were found in the south-southeastern regions of the country. A cold spell was considered to be when the cold days continued for at least two days at two or more nearby stations. Extreme and severe cold spells were also found to be maximum in the west-northwest Rajshahi and Rangpur divisions. More than 80% of cold-related mortality was seen all over Bangladesh during the calibration and validation periods when the average air temperature was less than or equal to 17 °C. The cold days on a 10-day scale showed that the highest 29.2% and 23.4% were seen all over the country during 11–20 January and 21–31 December, respectively. The significant increasing trend in cold days was found at four, three, and nine stations during 11–20 December, 21–31 December, and the month of December as a whole, respectively. Based on the above discussions, an average air temperature less than or equal to 17 °C can be used for the consideration of cold days and spells for operational purposes in Bangladesh.

## Figures and Tables

**Table 1:** Some example case studies that used the temperature anomalies method.

Ref.	Type of Temperature	Period *	Multiplier of SD	Region/Country	Description
[37]	*T_avg_*	1954–2005	1.5	China and Korea	Used 103 Chinese and 13 Korean weather stations. Assessed as a cold spell if it consecutively continued for at least 2 days.
[38]		1961–2012	1.5	Southeast China	Employed 100 grid points at a resolution of 0.5° × 0.5° in the surrounding area. Declared as a cold spell if persisted for at least 1–2 days.
[39]		1950–2005	1.5	USA	Data was used from the T62 Gaussian grid with 194 × 94 points. Used 237 grid points at a resolution of 1.25° × 0.9° longitude-latitude. If 10% of grid points exceeds considered extreme cold. Declared as an extreme cold spell if it persists for at least 3 or more days.
[40]		1979–2018	1.5	East Asia	ERA-Interim data is used for 1° × 1° resolution and considered cold when the air temperature drops more than one 5° × 5° box.
[33]		1975–2019	1.5	South Korea	Used 56 in situ stations. Temperature falls to 1.5 SD, which is approximately equal to 5.7 °C within 2 days.
[29]		1957/58–2000/01	1.5 and 2	South China	Used 172 Chinese and 5 Korean weather stations. Considered cold surges (if SD = 1.5) and strong ones (SD = 2) if the condition was consecutively sustained for 2 days.
[41]		1980–1999	2	Northern Hemisphere	Utilized seven global circulation model outputs with variable grid sizes. Assessed as a cold spell if consecutively continued for at least 2 days.
[42]		1951–2006	1.5 and 3	Europe	Employed at cell level with a resolution of 0.5° × 0.5°. Considered cold (if SD = 1.5) and very cold (SD = 3) if the condition was sustained over a few (undefined) consecutive days.
[43]	*T_max_*	1950–1972	1	Atlantic Canada	Used one weather station. Declared as a cold spell if the condition continued for at least 3 days.
[44]	*T_min_* and *T_max_*	1948–2016	1.25	Eastern USA	Employed 20 weather stations. Regarded as a cold spell if the condition was consecutively sustained for at least 5 days.

* Used to calculate the long-term average and its associated SD.

**Table 2:** Cold-related deaths in different divisions at different *T_avg_* for calibration and validation during the winters of 2009–2020, respectively.

	Temp. °C	Percent of Death in Different Divisions								
		Rangpur	Rajshahi	Mymensingh	Sylhet	Dhaka	Khulna	Barishal	Chattogram	Average
Calibration during 2009–2010 to 2014–2015	13	63	94	17	32	47	47	2		43
	14	86	98	79	32	62	55	4	12	54
	15	91	99	88	36	78	67	11	29	62
	16	99	99	88	44	84	79	43	31	71
	17	100	99	88	60	91	84	100	80	88
	18	100	100	88	100	91	84	100	81	93
	19	100	100	88	100	100	100	100	81	96
	20	100	100	100	100	100	100	100	100	100
Validation during 2015–2016 to 2020–2021	13	64	79	No deaths were found						72
	14	77	99		20		33			57
	15	80	100		37		72			72
	16	90	100		53	25	89	3	25	55
	17	97	100		70	67	100	100	75	87
	18	100	100		70	67	100	100	83	92
	19	100	100		100	92	100	100	83	96
	20	100	100		100	92	100	100	83	96
	21	100	100		100	100	100	100	100	100
Calibration during odd winter	13	73	97				32			67
	14	93	99	100	32	23	45	10	11	52
	15	94	99	100	63	54	58	40	76	73
	16	96	99	100	100	65	78	80	78	87
	17	99	99	100	100	85	81	100	78	93
	18	100	100	100	100	85	82	100	98	96
	19	100	100	100	100	96	100	100	100	100
	20	100	100	100	100	96	100	100	100	100
	21	100	100	100	100	100	100	100	100	100
Validation during even winter	13	44	95	44	19	68	53	1	94	52
	14	72	98	44	19	68	56	1	94	57
	15	83	100	67	22	68	79	1	94	64
	16	100	100	67	22	77	94	14	94	71
	17	100	100	67	47	87	100	100	94	87
	18	100	100	67	75	87	100	100	94	90
	19	100	100	67	100	100	100	100	100	96
	20	100	100	100	100	100	100	100	100	100

## Data Availability

All the output data have been available in the manuscript in the form of figures and tables.

## Supplementary Materials

The following is available online at https://www.mdpi.com/article/10.3390/s23052832/s1, Figure S1: Example case of calculating *T_avg_* using 1/2 hour, 1 h, and 3 hourly *T_a_* data; and daily *T_max_* and *T_min_* data during the 2018–2019 winter season at Dhaka University Automatic weather station.

## References

[1] Alam M.M., Mahtab A.S.M., Ahmed M.R., Hassan Q.K. (2022). Developing a Cold-Related Mortality Database in Bangladesh. Int. J. Environ. Res. Public Health.

[2] Vardoulakis S., Dear K., Hajat S., Heaviside C., Eggen B., McMichael A. (2014). Comparative assessment of the effects of climate change on heat-and cold-related mortality in the United Kingdom and Australia. Environ. Health Perspect..

[3] Massetti E., Mendelsohn R. (2015). How do heat waves, cold waves, droughts, hail and tornadoes affect US agriculture. CMCC Res. Paper.

[4] Si D., Jiang D., Lang X., Fu S. (2021). Unprecedented North American snowstorm and East Asian cold wave in January 2016: Critical role of the Arctic atmospheric circulation. Atmos. Sci. Lett..

[5] Rosselló J., Becken S., Santana-Gallego M. (2020). The effects of natural disasters on international tourism: A global analysis. Tour Manag..

[6] Budhathoki N.K., Zander K.K. (2019). Socio-economic impact of and adaptation to extreme heat and cold of farmers in the food bowl of Nepal. Int. J. Environ. Res. Public Health.

[7] Vajda A., Tuomenvirta H., Juga I., Nurmi P., Jokinen P., Rauhala J. (2014). Severe weather affecting European transport systems: The identification, classification and frequencies of events. Nat. Hazards.

[8] Añel J.A., Fernández-González M., Labandeira X., López-Otero X., De la Torre L. (2017). Impact of cold waves and heat waves on the energy production sector. Atmosphere.

[9] Kysely J., Pokorna L., Kyncl J., Kriz B. (2009). Excess cardiovascular mortality associated with cold spells in the Czech Republic. BMC Public Health.

[10] Guo Y., Jiang F., Peng L., Zhang J., Geng F., Xu J., Zhen C., Shen X., Tong S. (2012). The association between cold spells and pediatric outpatient visits for asthma in Shanghai, China. PLoS ONE.

[11] Ma W., Yang C., Chu C., Li T., Tan J., Kan H. (2013). The impact of the 2008 cold spell on mortality in Shanghai, China. Int. J. Biometeorol..

[12] Hwang S.W., Lebow J.M., Bierer M.F., O’Connell J.J., Orav E.J., Brennan T.A. (1998). Risk factors for death in homeless adults in Boston. Arch. Intern. Med..

[13] Vuillermoz C., Aouba A., Grout L., Vandentorren S., Tassin F., Moreno-Betancur M., Jougla É., Rey G. (2016). Mortality among homeless people in France, 2008–10. Eur. J. Public Health.

[14] Staddon P.L., Montgomery H.E., Depledge M.H. (2014). Climate warming will not decrease winter mortality. Nat. Clim. Chang..

[15] Stocker T. (2014). Climate Change 2013: The Physical Science Basis: Working Group I Contribution to the Fifth Assessment Report of the Intergovernmental Panel on Climate Change.

[16] Guo S., Yan D., Gui C. (2020). The typical hot year and typical cold year for modeling extreme events impacts on indoor environment: A generation method and case study. Build. Simul..

[17] Hu Y., He Y., Dong W. (2009). Changes in temperature extremes based on a 6-hourly dataset in China from 1961–2005. Adv. Atmos. Sci..

[18] Donaldson G., Ermakov S., Komarov Y.M., McDonald C., Keatinge W. (1998). Cold related mortalities and protection against cold in Yakutsk, eastern Siberia: Observation and interview study. BMJ.

[19] Xie H., Yao Z., Zhang Y., Xu Y., Xu X., Liu T., Lin H., Lao X., Rutherford S., Chu C. (2013). Short-term effects of the 2008 cold spell on mortality in three subtropical cities in Guangdong Province, China. Environ. Health Perspect..

[20] Huynen M.-M., Martens P., Schram D., Weijenberg M.P., Kunst A.E. (2001). The impact of heat waves and cold spells on mortality rates in the Dutch population. Environ. Health Perspect..

[21] Domonkos P., Kyselý J., Piotrowicz K., Petrovic P., Likso T. (2003). Variability of extreme temperature events in south–central Europe during the 20th century and its relationship with large-scale circulation. Int. J. Climatol..

[22] India Meteorological Department All India Multi-Hazard Winter Weather Warnings Bulletin. https://internal.imd.gov.in/section/nhac/dynamic/sigwxibf.pdf.

[23] Chen J., Yang J., Zhou M., Yin P., Wang B., Liu J., Chen Z., Song X., Ou C.-Q., Liu Q. (2019). Cold spell and mortality in 31 Chinese capital cities: Definitions, vulnerability and implications. Environ. Int..

[24] Sekhon N.S., Hassan Q.K., Sleep R.W. (2010). Evaluating potential of MODIS-based indices in determining “snow gone” stage over forest-dominant regions. Remote Sens..

[25] Hassan Q.K., Rahman K.M. (2013). Remote sensing-based determination of understory grass greening stage over boreal forest. J. Appl. Remote Sens..

[26] Sun R., Chen L.J.L., Planning U. (2012). How can urban water bodies be designed for climate adaptation?. Landsc. Urban. Plan..

[27] Karmakar S. (2019). Patterns of climate change and its impacts in northwestern Bangladesh. J. Eng. Sci..

[28] Khatun M.A., Rashid M.B., Hygen H.O. (2016). Climate of Bangladesh.

[29] Jeong J.H., Ho C.H. (2005). Changes in occurrence of cold surges over East Asia in association with Arctic Oscillation. Geophys. Res. Lett..

[30] Wheeler D., Harvey V., Atkinson D., Collins R., Mills M. (2011). A climatology of cold air outbreaks over North America: WACCM and ERA-40 comparison and analysis. J. Geophys. Res. Atmos..

[31] Heo J.-W., Ho C.-H., Park T.-W., Choi W., Jeong J.-H., Kim J. (2018). Changes in cold surge occurrence over East Asia in the future: Role of thermal structure. Atmosphere.

[32] Yong-Sang C., Chang-Hoi H., Gong D.-Y., Jeong J.-H., Park T.-W. (2009). Adaptive change in intra-winter distribution of relatively cold events to East Asian warming. TAO.

[33] Kim E.S., Ahn J.B. (2022). Study on the classification and characteristics of cold surge in South Korea. Int. J. Climatol..

[34] Park T.W., Ho C.H., Yang S., Jeong J.H. (2010). Influences of Arctic Oscillation and Madden-Julian Oscillation on cold surges and heavy snowfalls over Korea: A case study for the winter of 2009–2010. J. Geophys. Res. Atmos..

[35] Park T.-W., Ho C.-H., Deng Y. (2014). A synoptic and dynamical characterization of wave-train and blocking cold surge over East Asia. Clim. Dyn..

[36] Yang X., Zeng G., Zhang G., Iyakaremye V., Xu Y. (2021). Future projections of winter cold surge paths over East Asia from CMIP6 models. Int. J. Climatol..

[37] Park T.-W., Ho C.-H., Jeong S.-J., Choi Y.-S., Park S.K., Song C.-K. (2011). Different characteristics of cold day and cold surge frequency over East Asia in a global warming situation. J. Geophys. Res. Atmos..

[38] Ou T., Chen D., Jeong J.-H., Linderholm H.W., Zhou T. (2015). Changes in winter cold surges over southeast China: 1961 to 2012. Asia Pac. J. Atmos. Sci..

[39] Xie Z., Black R.X., Deng Y. (2017). The structure and large-scale organization of extreme cold waves over the conterminous United States. Clim. Dyn..

[40] Yang X., Zeng G., Zhang G., Li C.J.T., Climatology A. (2021). Linkage between interannual variation of winter cold surge over East Asia and autumn sea ice over the Barents Sea. Theor. Appl. Climatol..

[41] Vavrus S., Walsh J., Chapman W., Portis D. (2006). The behavior of extreme cold air outbreaks under greenhouse warming. Int. J. Climatol..

[42] Menzel A., Seifert H., Estrella N. (2011). Effects of recent warm and cold spells on European plant phenology. Int. J. Biometeorol..

[43] McCalla R., Day E., Millward H. (1978). The relative concept of warm and cold spells of temperature: Methodology and application. Arch. Meteorol. Geophys. Bioklimatol. B.

[44] Smith E.T., Sheridan S.C. (2018). The characteristics of extreme cold events and cold air outbreaks in the eastern United States. Int. J. Climatol..

[45] Mann H.B. (1945). Nonparametric tests against trend. Econometrica.

[46] Sen P.K. (1968). Estimates of the regression coefficient based on Kendall’s tau. J. Am. Stat. Assoc..

[47] (2020). Statistical Yearbook Bangladesh 2020.

[48] Islam N., Uyeda H. Comparison of TRMM 3B42 products with surface rainfall over Bangladesh. Proceedings of the IEEE International Geoscience and Remote Sensing Symposium.

[49] Bangladesh Geography. https://en.banglapedia.org/index.php?title=Bangladesh_Geography.

[50] Shahid S. (2009). Spatio-temporal variability of rainfall over Bangladesh during the time period 1969-2003. Asia Pac. J. Atmos. Sci..

[51] Alamgir M., Ahmed K., Homsi R., Dewan A., Wang J.-J., Shahid S. (2019). Downscaling and projection of spatiotemporal changes in temperature of Bangladesh. Earth Syst. Environ..

[52] Belvederesi C., Dominic J.A., Hassan Q.K., Gupta A., Achari G. (2020). Short-Term River Flow Forecasting Framework and Its Application in Cold Climatic Regions. Water.

[53] Belvederesi C., Dominic J.A., Hassan Q.K., Gupta A., Achari G. (2020). Predicting River Flow Using an AI-Based Sequential Adaptive Neuro-Fuzzy Inference System. Water.

[54] Abdullah A.Y.M., Bhuian M.H., Kiselev G., Dewan A., Hassan Q.K., Rafiuddin M. (2022). Extreme temperature and rainfall events in Bangladesh: A comparison between coastal and inland areas. Int. J. Climatol..

[55] Alam M.S., Liu Q., Schneider P., Mozumder M.M.H., Uddin M.M., Monwar M.M., Hoque M.E., Barua S. (2022). Stock Assessment and Rebuilding of Two Major Shrimp Fisheries (Penaeus monodon and Metapenaeus monoceros) from the Industrial Fishing Zone of Bangladesh. J. Mar. Sci. Eng..

[56] MMCH Fails to Provide Ambulance for Patients. https://www.thedailystar.net/country/mmch-fails-provide-ambulance-patients-1211965.

[57] Baten N., Hossain M.A., Rahman M.H., Rahman M.A. (2022). Cold wave condition over Bangladesh for the period of 1988–2017. Dew Drop..

[58] Proximity to Water Bodies. https://www.acer-acre.ca/resources/climate-change-in-context/general-concepts/proximity-to-water-bodies#:~:text=Large%20bodies%20of%20water%20such,cooler%20and%20in%20winter%20warmer.

[59] Rahman M., Lateh H. (2016). Spatio-temporal analysis of warming in Bangladesh using recent observed temperature data and GIS. Clim. Dyn..

[60] Mullick M.R.A., Nur R.M., Alam M.J., Islam K.A. (2019). Observed trends in temperature and rainfall in Bangladesh using pre-whitening approach. Glob. Planet. Chang..

[61] Imran H., Kala J., Uddin S., Islam A.S., Acharya N. (2023). Spatiotemporal analysis of temperature and precipitation extremes over Bangladesh using a novel gridded observational dataset. Weather. Clim. Extrem..

