# Supplementary material for: Characterizing Cold Days and Spells and Their Relationship with Cold-Related Mortality in Bangladesh
Source: Sensors (Basel). 2023 Mar 5;23(5):2832. doi: 10.3390/s23052832 (PMC10007433; doi:10.3390/s23052832)
Supplement: Supplementary file 1 [file sensors-23-02832-s001.zip › sensors-2162075-supplementary.pdf]

Supplementary Information

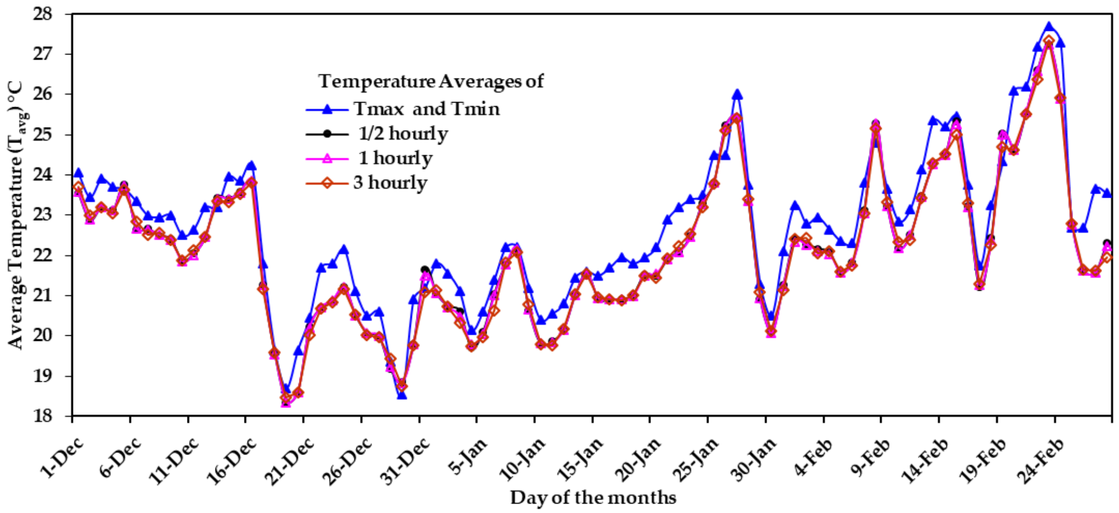

Figure S1: Example case of calculating T_{avg} using ½ hour, 1 hour, and 3 hourly T_a data; and daily T_{max} and T_{min} data during the 2018-2019 winter season at Dhaka University Automatic weather station.
